# Metal sensitivity in total joint arthroplasty: None of the current diagnostic tests are reliable, sensitive and specific enough to guide treatment decisions!

**DOI:** 10.1002/ksa.12706

**Published:** 2025-06-08

**Authors:** Umile Giuseppe Longo, Giovanni Intermesoli, Raffaele Di Tommaso, Alberto Lalli, Bruno Violante, Michael T. Hirschmann

**Affiliations:** ^1^ Fondazione Policlinico Universitario Campus Bio‐Medico Rome Italy; ^2^ Orthopaedic Department Clinical Institute Sant'Ambrogio, IRCCS ‐ Galeazzi Milan Italy; ^3^ Department of Orthopedic Surgery and Traumatology Kantonsspital Baselland Bruderholz Switzerland; ^4^ Department of Clinical Research, Research Group Michael T. Hirschmann Regenerative Medicine & Biomechanics University of Basel Basel Switzerland

**Keywords:** allergy, hypersensitivity, joint arthroplasty, metals, nickel, nickel‐free

## Abstract

**Purpose:**

This systematic review was conducted to evaluate the current literature on metal hypersensitivity in patients undergoing joint arthroplasty. The aims of the study were to report diagnostic tools used to assess metal hypersensitivity and to report complications arising in patients who are hypersensitive to nickel or other metals performing joint arthroplasty. Given the potential impact on implant longevity and patient outcomes, understanding the clinical relevance of metal hypersensitivity is crucial for optimising surgical decision‐making.

**Methods:**

This systematic review adheres to PRISMA guidelines and evaluates the variability in diagnostic approaches and the challenges in clinical management. Included in this review were studies involving patients sensitive to nickel or other metals undergoing joint arthroplasty. Eligibility criteria focused on commonly employed diagnostic tools and associated complications. A comprehensive literature search was conducted across Medline, EMBASE, Scopus, CINAHL, and CENTRAL databases. The methodological quality of the included studies was assessed using the Joanna Briggs Institute Critical Appraisal tool for case series and the ROBINS‐I tool for case‐control studies.

**Results:**

Twenty‐four articles met the inclusion criteria and were included in this systematic review. A total of 4865 patients undergoing joint arthroplasty were selected. Diagnostic tools included patch testing, lymphocyte transformation test (LTT), and medical history assessment. Variability in time point of testing and diagnostic protocols was noted. Complications including joint pain, swelling, reduced range of motion, and implant failure were reported in 12 studies. Clinical outcomes varied widely: some studies showing no significant differences between hypersensitive and non‐hypersensitive patients, while others reported increased pain and reduced joint function.

**Conclusion:**

There is a lack of a standardised protocol for diagnosing metal hypersensitivity, leading to uncertainty regarding test selection and timing. This inconsistency leads to variability in reported outcomes, with limited studies focusing on post‐surgical hypersensitivity in patients.

**Level of Evidence:**

Level III, systematic review.

**Abbreviations**
CoCr: cobalt chromium
CoCrMo: cobalt chromium molybdenum
CoCrNi: cobalt chromium nickel
FJS‐12: forgotten joint score
KOOS: knee osteoarthritis outcome score
KSC: knee society clinical
KSF: knee society functional
KSP: knee society pain
LEAS: lower extremity activity scale
LOS: length of stay
LTT: lymphocyte transformation testing
OKS: oxford knee score
OxZi/Ti: oxidised zirconium and titanium
PCC: prospective case‐control
PCS: prospective case‐series
PI‐NRS: pain intensity numerical rating scale
PRISMA: preferred reporting items for systematic reviews and meta‐analyses
PROMIS10: patient‐reported outcomes measurement information system
RCC: retrospective case‐control
RCS: retrospective case series
RCT: randomised control trial
ROBINS‐I: risk of bias in non‐randomised studies‐of interventions
ROM: range of motion
THA: total hip arthorplasty
TiAlV: titanium aluminium vanadium
TiNbN: titanium niobium nitride
TJR: total joint replacement
TKA: total knee arthroplasty
TSA: total shoulder arthroplasty
UCLA: university of california
UHMWPE: ultrahigh molecular weight polyethylene
UKA: unicondylar knee arthroplasty
VAS: visual analog scale
VR12: veterans rand 12‐item component survey

## INTRODUCTION

The prevalence of metal hypersensitivity in the general population ranges from 10% to 15% [23]. Nickel hypersensitivity is the most common [2], with an estimated prevalence of cutaneous sensitivity to nickel in the general population of 14% [4, 17, 27].

The literature has extensively discussed the issue of hypersensitivity to implanted metallic orthopedic devices and its implications, being a topic of significant debate [1]. Joint replacement implants are mostly made of stainless steel, containing nickel (13%–15%) and chromium (17%–19%).

The recorded prevalence of metal sensitivity in total hip arthroplasty (THA) patients with well‐functioning implants is 25%, and 60% in those with failed or poorly functioning implants [17]. Similarly, in total knee arthroplasty (TKA) the prevalence is reported to be 44% in patients with stable implants, reaching up to 57% in case of loosened implants [14]. Therefore, some authors have reported that metal sensitivity might contribute to implant failure [15, 17]. Despite these findings, the direct causal relationship between metal hypersensitivity and implant failure remains uncertain, as other factors such as mechanical wear, infection and aseptic loosening also contribute to implant‐related complications [17].

To date, there is no established test for identifying metal hypersensitivity as a contributor to implant failure. Skin patch testing is generally accepted for its ease of application, availability, comprehensive evaluation, and prompt results [29, 41]. However, its effectiveness, along with lymphocyte transformation testing (LTT), has faced scrutiny [32] and debates persist on whether individuals without a documented history of metal hypersensitivity reactions require screening before implantation [5, 33]. A different perspective proposes that relying only on the patient's personal history of metal reactions is not adequately predictive to justify patch testing. Consequently, the indication for skin patch testing prior to orthopedic implantation is still under debate [33]. Furthermore, studies have shown that patch testing may not reliably predict in vivo reactions to implanted metal components, raising concerns about its clinical utility [32, 33]. The LTT faces challenges in widespread clinical use due to accessibility, lack of standardisation, inter‐laboratory variability, and limited insurance coverage [41]. The exact role of the LTT remains uncertain, but appears to be gaining support for combined use with the patch test and potentially in association with peri‐implant histopathology [32]. Currently, it is a common consensus that none of those tests are reliable, sensitive and specific enough to base treatment decisions on these. A standardised diagnostic protocol is needed to improve preoperative assessment and postoperative management of patients suspected of metal hypersensitivity [1].

This systematic review was conducted to evaluate the current literature on metal hypersensitivity in patients undergoing joint arthroplasty. The aims of the study were to analyse the diagnostic accuracy of various testing methods for metal hypersensitivity, including patch testing, LTT, and medical history assessment and to determine the clinical impact of metal hypersensitivity on postoperative complications such as pain, implant failure, and revision surgery. We hypothesise that current diagnostic tools demonstrate inconsistencies in predicting clinical outcomes, and that metal hypersensitivity contributes to postoperative complications, though its role as a primary cause of implant failure remains controversial.

**Clinical Relevance:** Understanding the relationship between metal hypersensitivity, diagnostic accuracy, and implant‐related complications is crucial for optimising patient selection and implant choice. Given the ongoing debate regarding preoperative testing and its utility, this study aims to clarify whether screening for metal hypersensitivity should become a standard part of preoperative assessment or if its clinical impact is too limited to justify routine testing.

## MATERIALS AND METHODS

**Figure 1:**
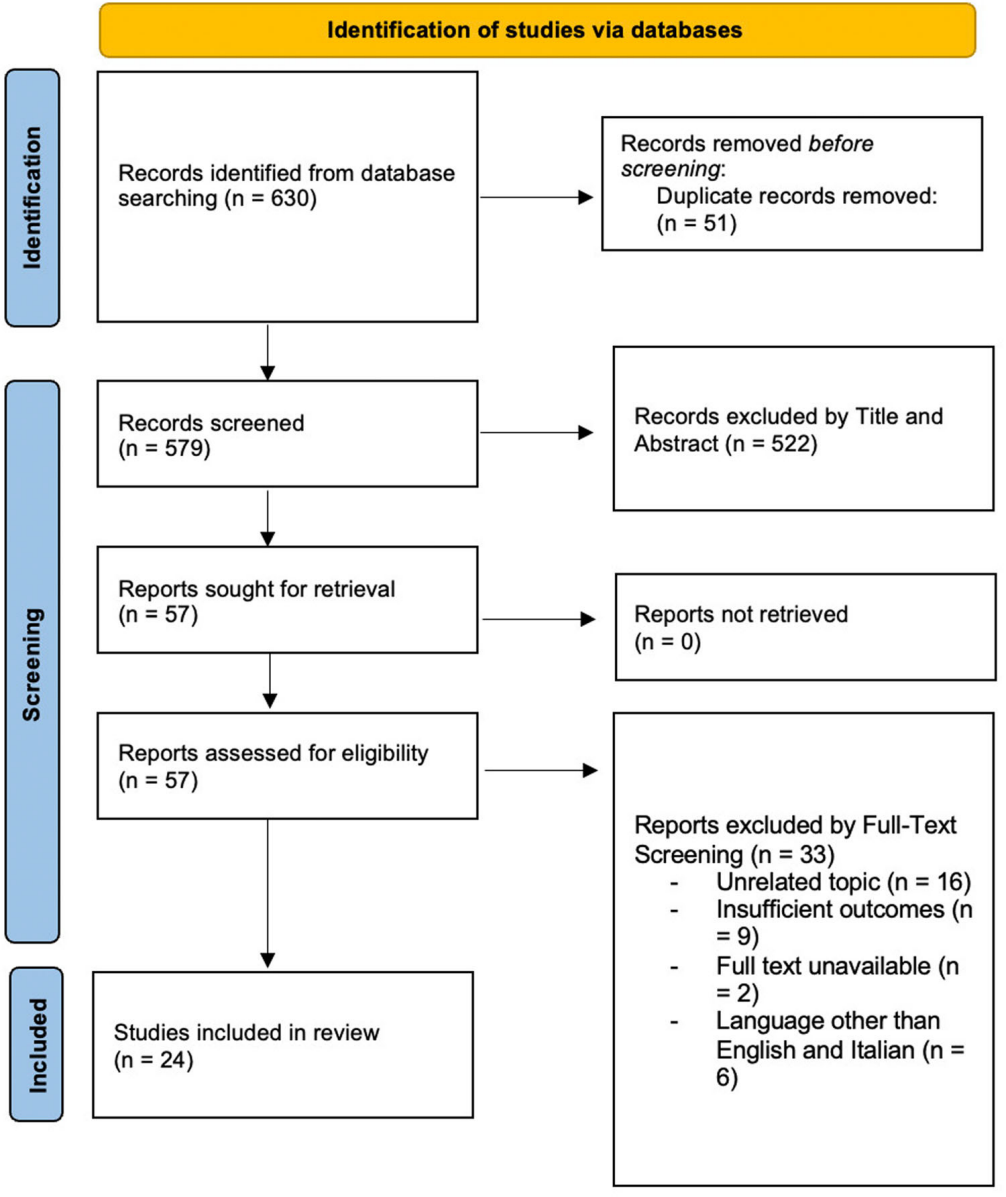
PRISMA flowchart.

Two independent reviewers separately performed the article screening. All papers were initially screened for relevance by title and abstract, followed by full‐text screening. The two investigators then performed data extraction. Finally, the included papers were discussed among the authors. In case of disagreement between investigators, a third investigator was responsible to make the final decision. The selection process was reported in Figure 1.

### Literature search

A systematic review was performed using the Preferred Reporting Items for Systematic Reviews and Meta‐analyses (PRISMA) guidelines [31]. A comprehensive search from inception to November 2024 was performed on Medline, EMBASE, Scopus, CINAHL and CENTRAL bibliographic databases using the following string: (nickel free) AND (prosthesis), (total knee arthroplasty) OR (total knee replacement) AND [46] OR (nickel free), (hip arthroplasty) OR (hip replacement) AND [46], (metal sensitivity) [38] AND (replacement)) AND (nickel free), (arthroplasty) OR (replacement)) AND (nickel free), (arthroplasty) OR (replacement)) AND (metal sensitivity)) AND [11, 46], AND (nickel free) [46], AND (prosthesis) AND (arthroplasty) AND (allergy) [38]. Keywords were used both isolated and combined. Further studies were sought by examining the reference lists of selected papers and systematic reviews.

### Eligibility criteria

The following study designs were considered for eligibility: randomised control trials (RTCs), prospective case‐control studies (PCC), prospective case series (PCS), retrospective case‐control studies (RCC), and retrospective case series (RCS). Given the author's proficiency in multiple languages, papers in English and Italian were screened. Peer‐reviewed articles of each level of evidence according to Oxford classification were considered.

Included in this review were studies involving patients sensitive to nickel or other metals undergoing joint arthroplasty. The eligibility criteria centred on studies that specifically focus on commonly used diagnostic tools for evaluating metal hypersensitivity. Additionally, the selected studies were required to address potential complications arising post‐implantation due to metal hypersensitivity.

Excluded from consideration in this review were technical notes, letters to editors, instructional courses, or studies that primarily focused on potential complications due to metal hypersensitivity arising in the fields other than orthopaedics. In vitro, animal, cadaver and biomechanical studies were excluded.

### Outcomes of interest

Author, year of publication, type of study, level of evidence (LoE), sample size, number of patients, mean age, gender, type of intervention, type of hypersensitivity test, timing of testing, number of allergic patients, and nature of allergies were extracted for analysis.

Clinical outcomes assessed included the University of California (UCLA) activity scale, the Knee Society Clinical (KSS‐C) score, the Knee Society Functional (KSS‐F) score, the Knee Society Pain (KSS‐P) score, the Knee Osteoarthritis Outcome Score (KOOS), the Visual Analog Scale (VAS), the Lower Extremity Activity Scale (LEAS), the Patient‐Reported Outcomes Measurement Information System (PROMIS, the Veterans RAND 12‐item (VR12) Component Survey, the Forgotten Joint Score (FJS‐12), length of stay (LOS), the Oxford Knee Score (OKS), patients’ satisfaction, Pain Intensity Numerical Rating Scale (PI‐NRS), range of motion (ROM) and complications.

Only outcome measures at latest follow‐up were analysed, including mean values and standard deviation, when calculated.

### Methodological quality assessment

The methodological quality of each study was assessed by two independent reviewers. A third reviewer was consulted in case of discrepancies. Considering the design of the included studies, the Joanna Briggs Institute Critical Appraisal tool for case series [31], and the Risk of Bias In Non‐randomised Studies‐of Interventions (ROBINS‐I) tool for case‐control studies were selected to assess the methodological quality of each paper [39].

## RESULTS

### Study selection

A total of 630 articles were identified, of which 51 were excluded after duplicate removal. Of 579 remaining articles, 522 were rejected during title and abstract screening and 33 were rejected during full‐text screening. Finally, twenty‐four articles met the inclusion criteria and were included in this systematic review. Figure 1 displays the PRISMA flowchart.

### Quality of evidence

**Figure 2:**
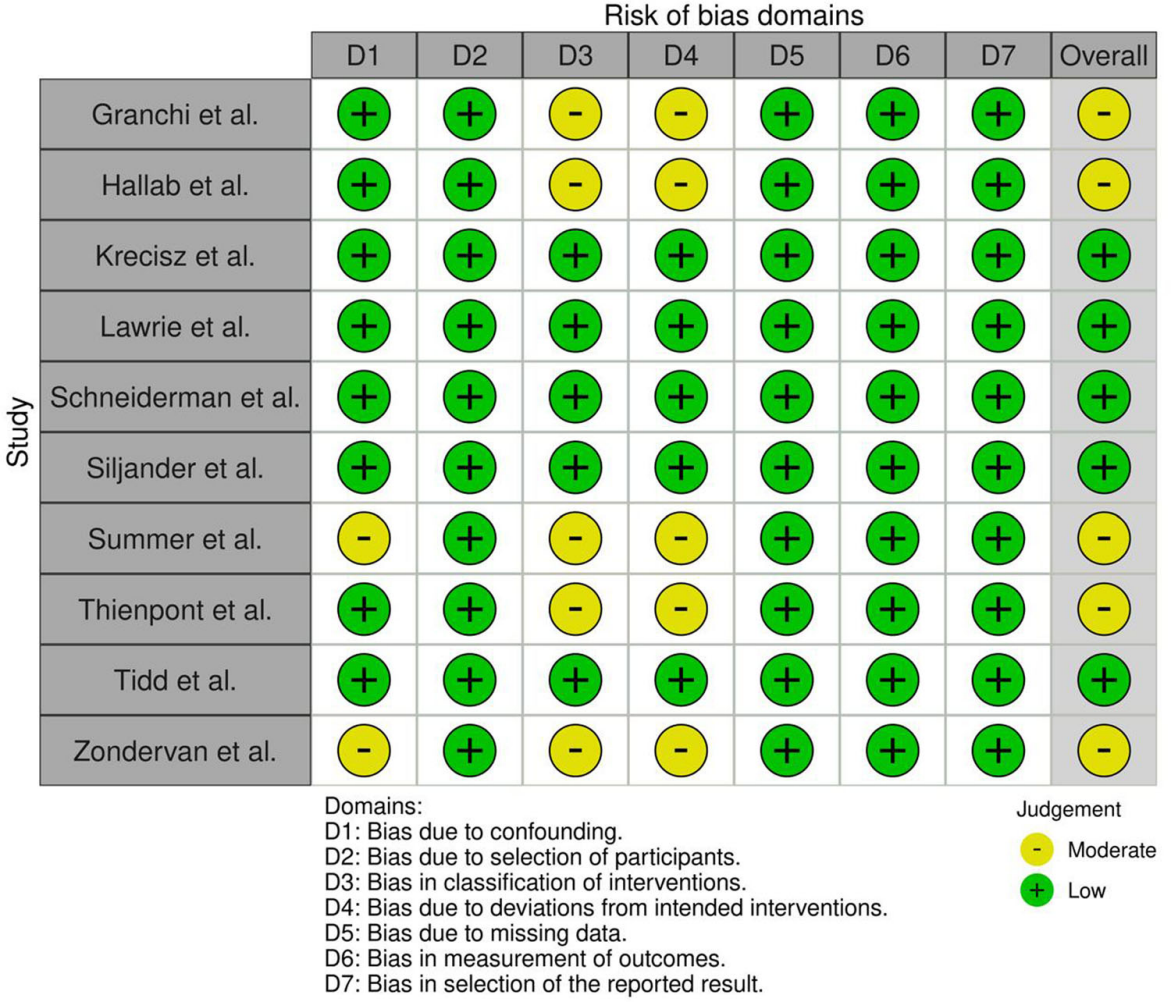
Risk of bias assessment for non‐randomised case‐control trials (ROBINS‐I).

**Figure 3:**
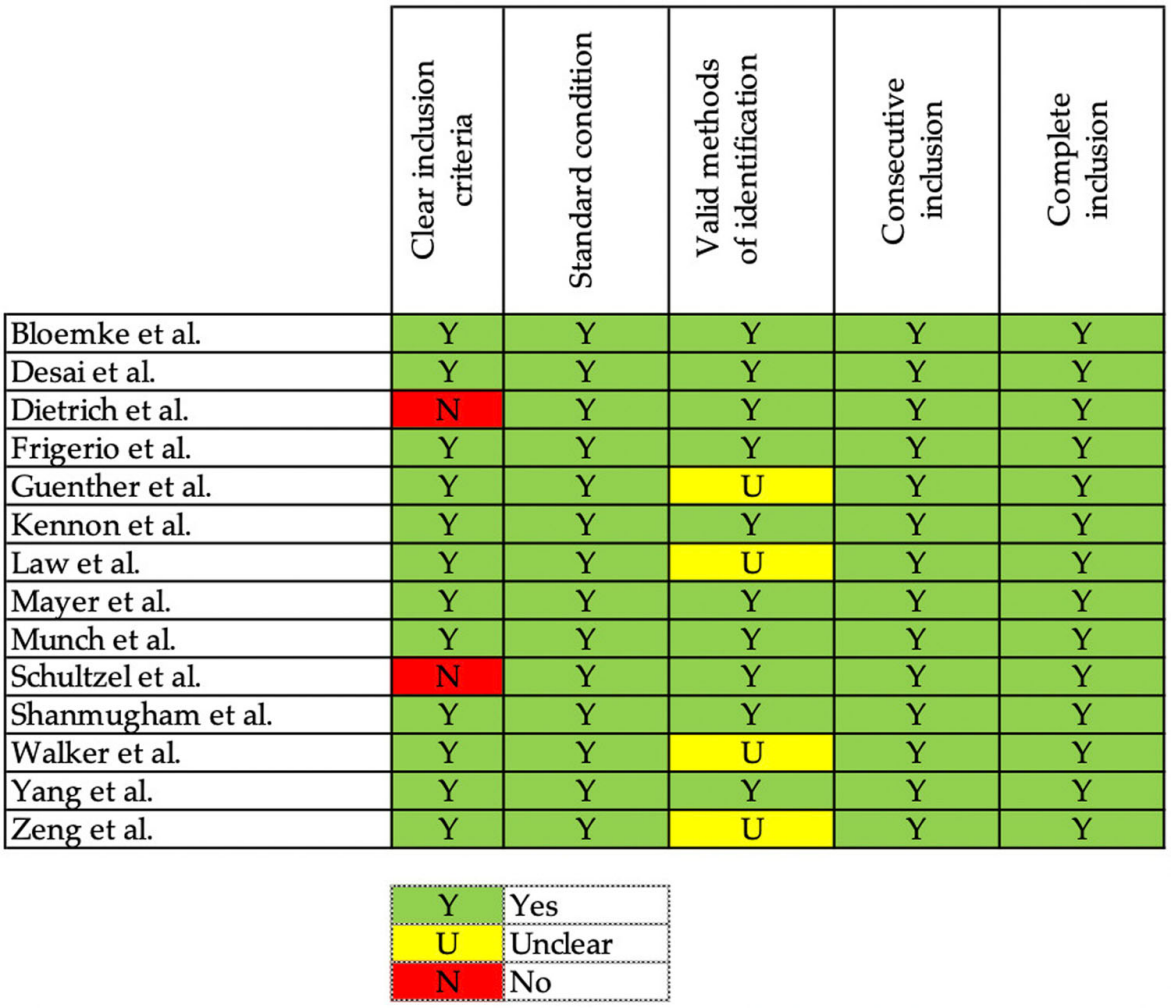
Risk of bias assessment for case series studies.

Case control studies were judged as “low risk of bias” [22, 25, 34, 37, 45] or “moderate risk of bias” [15, 18, 40, 43, 51]. The mean quality of case‐series studies was overall good [4, 8, 9, 12, 16, 21, 24, 28, 30, 35, 36, 47, 49, 50]. The methodological quality assessment for CCs and CSs are shown in Figures 2, 3, respectively.

### Study characteristics

The 24 papers deemed eligible for inclusion in the present review were published between 2006 and 2024. The following studies were included: eight RCC studies with LoE of III [15, 18, 34, 37, 40, 43, 45, 51], two PCC studies with LoE of III [22, 25], 10 RCS studies with LoE of IV [4, 9, 16, 21, 24, 30, 35, 36, 47, 49] and four PCS studies with LoE of IV [8, 12, 28, 50].

A total of 4865 patients undergoing the following procedures were selected: TKA, revision TKA, THA, revision THA, total shoulder arthroplasty (TSA) and unicondylar knee arthroplasty (UKA).

Study characteristics and sample sizes are shown in Table 1.

**Table 1:** Study characteristics.

Author	Year	Study design; LoE	Journal	No of patients	Mean age	Gender (M; F)
Bloemke et al.	2015	RCS; IV	*Journal of Knee Surgery*	139	NR	53; 86
Desai et al.	2019	PCS; IV	*Journal of Orthopaedics*	233	59.59 (30–78)	84; 149
Dietrich et al.	2009	RCS; IV	*Journal der Deutschen Dermatologischen Gesellschaft*	4	63.5 (57–68)	0; 4
Frigerio et al.	2011	PCS; IV	*Contact Dermatitis*	100	68	27; 73
Granchi et al.	2006	RCC; III	*Journal of Biomedical Materials Research*	223	Group A: 59.6 ± 13 Group B: 65.0 ± 11 Group C: 64.7 ± 11	57; 161
Guenther et al.	2015	RCS; IV	*International Orthopaedics*	855	NR	63; 792
Hallab et al.	2008	RCC; III	*Journal of Orthopaedic Surgery and Research*	31	Group 1: 29 (range 23–36) Group 2: 69 (range 55–80) Group 3: 70 (range 49–80)	15; 16
Kennon et al.	2020	RCS; IV	*Journal of Shoulder and Elbow Surgery*	43	70.4 (52–87)	10; 33
Krecisz et al.	2012	PCC; III	*International Journal of Occupational Medicine and Environmental Health*	60	61.7	17; 43
Law et al.	2020	RCS; IV	*Arthroplasty Today*	346	64.7 (36–89)	18; 328
Lawrie et al.	2022	PCC; III	*The Journal of Arthroplasty*	40	OxZi/Ti group: 66.75 ± 7.92 CoCr group: 62.6 ± 10.02	20; 20
Mayer at al.	2021	PCS; IV	*The Journal of Allergy and Clinical Immunology*	105	61.4 (33–83)	47; 58
Münch et al.	2015	RCS; IV	*Acta Orthopaedica*	327	NR	93; 234
Schneiderman et al.	2021	RCC; III	*The Journal of Arthroplasty*	22	NR	10; 12
Schultzel et al.	2020	RCS; IV	*The Permanente Journal*	840	NR	NR
Shanmugham et al.	2021	RCS; IV	*Indian Journal of Dermatology, Venereology and Leprology*	30	55.0 ± 13.7	16; 14
Siljander et al.	2023	RCC; III	*The Journal of Arthroplasty*	282	CoCr group: 70 (49–83) Nickel‐free group: 65 (30–86)	8; 274
Summer et al.	2010	RCC; III	*Contact Dermatitis*	20	56.36 ± 15.20	6; 14
Thienpont et al.	2023	RCC; III	*Archives of Orthopaedic and Trauma Surgery*	154	Group 1: 75 ± 9 Group 2: 68 ± 9	27; 127
Tidd et al.	2024	RCC; III	*The Journal of Arthroplasty*	760	Hypoallegenic group: 66.6 (44‐84) Standard group: 66.4 (28–88)	20; 740
Walker et al.	2019	RCS; IV	*The Bone and Joint Journal*	82	64.3 (39–87)	5; 77
Yang et al.	2019	RCS; IV	*The Journal of Bone and Joint Surgery*	27	64.0 ± 11.7 (36–87)	6; 21
Zeng et al.	2014	PCS; IV	*International Orthopaedics*	96	THA group: 48.28 ± 14.87 TKA group: 65.07 ± 9.16	39; 57
Zondervan et al.	2019	RCC; III	*Journal of Orthopaedic Surgery and Research*	46	61.47 ± 9.34	19; 27

Abbreviations: CoCr, cobalt chromium; LoE, level of evidence; NR, not reported; OxZi/Ti, oxidised zirconium and titanium; PCC, prospective case–control; PCS, prospective case‐series; RCC, retrospective case–control; RCS, retrospective case series; THA, total hip arthroplasty; TKA, total knee arthroplasty.

### Patient characteristics

Sixteen studies included patients undergoing TKA [4, 8, 9, 12, 16, 22, 24, 25, 28, 30, 36, 37, 40, 43, 45, 50], nine studies patients undergoing THA [12, 15, 16, 18, 22, 28, 36, 40, 50], five studies patients undergoing revision TKA [16, 30, 34, 49, 51], one study patients undergoing revision THA [16], three studies patients undergoing TSA [21, 28, 36], and one study assessed patients undergoing UKA [47]. One paper did not report the type of joint replacement procedure performed [35].

Two studies included patients not receiving any implant as control groups [18, 40].

The number of allergic patients was recorded in every paper included in the present review, with twenty papers describing the nature of allergies (i.e., metals to which study participants are allergic) [8, 9, 12, 15, 16, 18, 21, 22, 25, 28, 30, 34, 35, 36, 37, 40, 47, 49, 50, 51]. The metallic composition of the implants used for joint arthroplasty was recorded in thirteen articles [9, 12, 15, 18, 24, 25, 34, 37, 43, 45, 47, 49, 51].

The results, including features of patient groups of each study, are shown in Table 2.

**Table 2:** Patients characteristics.

Author	Bloemke et al.	Desai et al.	Dietrich et al.	Frigerio et al.	Granchi et al.	Guenther et al	Hallab et al.
Intervention	TKA	TKA	TKA	TKA, THA	THA	TKA, rTKA, THA, rTHA	THA
No of patients	139	233	4	100	223	855	31
Patient group	TKA patients assessed postoperatively	TKA patients assessed postoperatively	TKA patients assessed postoperatively	TKA patients assessed pre‐ and post operatively (52) THA patients assessed pre‐ and post operatively (48)	Group A: THA patients assessed preoperatively (66) Group B: THA patients with stable prosthesis assessed postoperatively (53) Group C: THA patients with loose prosthesis assessed postoperatively (104)	TKA, rTKA, THA, rTHA patients assessed preoperatively	Group 1: young healthy subjects without metallic implants (8) Group 2: THA patients with well functioning implants (15) Group 3: age‐matched controls to Group 2 with no implants (8)
No allergic/sensitised patients	20	37	4	37 (16 preoeratively, 21 postoperatively)	93 (Group A: 26; Group B: 27; Group C: 40)	855	7
Nature of allergy/sensitivity	NR	Chromium sensitivity in 27 patients (11.58%) Nickel sensitivity in 20 patients (8.58%) Cobalt sensitivity in 15 patients (6.43%)	Nickel allergy in 4 patients (100%) Cobalt allergy in 2 patients (50%)	Nickel sensitivity in 21 patients (21%) Cobalt sensitivity in 8 patients (8%) Palladium sensitivity in 3 patients (3%)	93 patients (41.70%) sensitive to at least one of the following haptens: nickel sulphate, chromium, chobalt chloride, ferric chloride, molybdenum chloride, manganese chloride, titanium dioxide, aluminium chloride, vanadium trichloride	Nickel sensitivity in 849 patients (99.29%) Cobalt sensitivity in 84 patients (9.82%) Chromium sensitivity in 39 patients (4.56%) Cement component sensitivity in 14 patients (1.63%)	Chromium sensitivity in 5 patients (16.12%) Nickel sensitivity in 1 patient (3.22%) Cobalt sensitivity in 1 patient (3.22%) Cobalt sensitivity in 2 patients (6.45%)
Type of implant	NR	NR	1 patient: Cement‐free CoCrMo‐based endoprosthesis 1 patient: CoCrMo‐based endoprosthesis with partial cementing 1 patient: CoCrMo‐based medial endoprosthesis 1 patient: NR	THA 24 patients: Acetabular cup CoCrMo; Femoral stem TiAlV 14 patients: TiAlVi 10 patients: CoCrMo TKA 33 patients: Femoral component CoCrMo; Tibial component	Group A: ‐ Group B: 2 patients CoCrMo; 27 patients TiAlV; 24 patients CoCrMo/TiAlV Group C: 31 patients CoCrMo; 25 patients TiAlV; 22 patients CoCrMo/TiAlV; 26 patients Uknown	NR	Group 1: ‐ Group 2: TiAlV stem, CoCrMo head, UHMWPE acetabular cup in TiAlV lining Group 3: ‐

Author	Kennon et al.	Kręcisz et al.	Law et al.	Lawrie et al.	Mayer et al.
Intervention	TSA	TKA, THA	TKA	TKA	TKA, THA, TSA
No of patients	43	60	346	40	05
Patient group	TSA patients with self‐reported allergy (31) TSA patients with self‐reported allergy that performed patch testing (12)	Stage I: TKA and THA patients assessed preoperatively (60) Stage II: TKA and THA patients assessed postoperatively (48)	TKA patients with self‐reported metal allergy	OxZr/Ti group: patients with self‐reported nickel allergy undergoing TKA using nickel‐sensitive implant (16) CoCr group: patients without self‐reported nickel allergy undergoing TKA using non‐hyopoallergenic implant (24)	TKA, THA, TSA patients with implant failure assessed postoperatively for evaluation of implant sensitisation as a cause of failure
No allergic/sensitised patients	43	Stage I: Self‐reported allergy: 13; Positive patch test: 13 Stage II: Positive patch test: 12	346	16	61
Nature of allergy/sensitivity	Self reported allergy: Nickel allergy in 37 patients (86.04%) Cobalt allergy in 4 patients (9.30%) Chromium allergy in 4 patients (9.30%) Copper allergy in 2 patients (4.65%) Gold allergy in 2 patients (4.65%) Zinc allergy in 1 patient (2.33%) Titanium allergy in 1 patient (2.33%) Nonspecific metal allergy in 8 patients (18.60%) Patch test: Nickel allergy in 12 patients (27.90%) Cobalt allergy in 2 patients (4.65%) Chromium allergy in 1 patient (2.33%)	Stage I: Nickel sulphate allergy in 12 patients (20%) Palladium chloride allergy in 8 patients (13.33%) Cobalt chloride allergy in 6 patients (10%) Potassium dichromate allergy in 3 patients (5%) Stage II: Nickel sulphate allergy in 10 patients (16.66%) Palladium chloride allergy in 5 patients (8.33%) Cobalt chloride allergy in 8 patients. (13.33%) Potassium dichromate allergy in 4 patients (6.66%) Vanadium chloride allergy in 1 patient (1.66%)	NR	Nickel allergy in 16 patients (40%)	Nickel sensitisation in 25 patients (23.80%) Cobalt sensitisation in 16 patients (15.23%) Chromium sensitisation in 1 patient (0.95%) Titanium sensitisation in 1 patient (0.95%) Bone cement sensitisation in 39 patients (37.14%)
Type of implant	NR	NR	346 patients: alternative bearing ion‐bombarded titanium implant	16 patients: OxZr/Ti 24 patients: CoCr	NR

Author	Münch et al	Schneiderman et al.	Schultzel et al.	Shanmugham et al.	Siljander et al.	Summer et al.
Intervention	TKA, rTKA	rTKA	TJR	TKA, THA, TSA	TKA	TKA, THA
No of patients	327	22	840	30	282	20
Patient group	TKA, rTKA patients assessed preoperatively (253) and postoperatively (74)	Group A: rTKA patients with negative preoperative LTT (6) Group B: rTKA patients with positive preoperative LTT for nickel (12) Group C and D: LTT‐positive patients who had undergone a first revision to a hypoallergenic implant for possible immune failure of a conventional (CoCrNi) primary TKA and subsequently underwent rerevision. These patients, before and after rerevision, comprise the cases in group C and group D, respectively (4)	TJR patients assessed postoperatively	TKA, THA, TSA patients assessed preoperatively and postoperatively	TKA patients with self‐reported preoperative nickel allergy receiving nickel‐free implants (243) and CoCr implants (39)	Group 1: Patients without Nickel allergy and without metal implants (5) Group 2: Patiens with Nickel allergy and without metal implants (5) Group 3: Patients with Nickel allergy and with well‐functioning TKA (5) Group 4: Patients Nickel allergy and with TKA‐ or THA‐related sympotms (5)
No allergic/sensitised patients	50	16	Self‐reported allergy: 41 Positive patch test: 34	NR	282	15
Nature of allergy/sensitivity	Nickel allergy in 39 patients (11.92%) Cobalt allergy in 9 patients (2.75%) Chromium allergy in 12 patients (3.66%)	Nickel sensitivity in 16 patients (72.72%) Cobalt sensitivity in 4 patients (18.18%) Chromium sensitivity in 3 patients (13.6%)	Nickel sensitivity in 27 patients (3.21%) Cobalt sensitivity in 4 patients (0.47%) Gold sensitivity in 4 patients (0.47%) Tin sensitivity in 1 patient (0.11%) Chromium sensitivity in 1 patient (0.11%) Titanium sensitivity in 1 patient (0.11%)	Preoperative patch test: nickel sulphate sensitivity in 3 patients (10%), potassium dichromate sensitivity in 1 patient (3.33%) Postoperative patch test: nickel sulphate sensitivity in 3 patients (10%), cobalt chloride sensitivity in 2 patients (6.66%)	Nickel allergy in 282 patients (100%)	Nickel allergy in 15 patients (75%)
Type of implant	NR	Group A: NR Group B: 11 patients titanium; 1 patient ceramic‐coated Group C: 3 patients titanium; 1 patient ceramic‐coated Group D: 3 patients ceramic‐coated; 1 patient titanium	NR	NR	243 patients: nickel‐free implants (metallic composition NR) 39 patients: CoCr	NR

Author	Thienpont et al.	Tidd et al.	Walker et al.	Yang et al.	Zeng et al.	Zondervan et al.
Intervention	TKA	TKA	UKA	rTKA	TKA, THA	rTKA
No of patients	154	760	82	27	96	46
Patient group	Group 1: TKA partients with self‐reported preoperative cobalt, chrome or nickel allergy receiving TiNbN implant (51) Group 2: TKA partients without preoperative cobalt, chrome or nickel allergy receiving CoCr implant (103)	Hypoallergenic group: TKA patients with documented metal allergy receiving hypoallergenic implants Standard group: TKA patients without documented metal allergy receiving standard implants	UKA patients reporting cutaneous symptoms of metal hypersensitivity to nickel, cobalt, chromium receiving standard CoCr implants	Patients with well‐fixed primary TKA implants and persistent pain and/or stifness that underwent revision due to a suspected metal	THA group: patients undergoing THA with metal allergy (metal allergy group (36)) and without metal allergy (non‐metal allergy group (26)) assessed preoperatively TKA group: patients undergoing TKA with metal allergy (metal allergy group (11)) and without metal (non‐metal allergy group (14)) assessed preoperatively	Reactive group: patients with painful primary TKA revised to hypoallergenic implants if tested positive for metal LTT sensitivity Non‐reactive group: patients with painful primary TKA revised for arthrofibrosis if tested negative for metal LTT sensitivity
No allergic/sensitised patients	51	199	82	27	47 (36 patients in THA group, 11 patients in TKA group)	39
Nature of allergy/sensitivity	NR	NR	Single nickel hypersensitivity in 68 patients (82.92%) Nickel and cobalt hypersensitivity in 10 patients (12.19%) Nickel and chromium hypersensitivity in 4 patients (4.87%)	Nickel sensitivity in 27 patients (100%) Cobalt sensitivity in 4 patients (14.81%) Chromium sensitivity in 5 patients (18.51%)	Nickel, cobalt, manganese, and tin were the most common allergic metal elements	Nickel sensitivity was the most common, found in 32 patients (69.56%)
Type of implant	Group 1: TiNbN Group 2: CoCr	Hypoallergenic group: nickel‐free implants (metallic composition NR) Standard group: standard implants (metallic composition NR)	82 patients: CoCr	27 patients: nickel‐free implants (25 patients custom titanium; 1 patient niobium‐coated CoCr; 1 patient zirconia oxide)	NR	Reactive group: hypoallergenic implants (metallic composition NR) Non‐reactive group: standard implants (metallic composition NR)

Abbreviations: CoCr, cobalt chromium; CoCrMo, cobalt chromium molybdenum; CoCrNi, cobalt chromium nickel; LTT, lymphocyte transformation testing; NR, not reported; OxZi/Ti, oxidised zirconium and titanium; rTKA, revision total knee arthroplasty; rTHA, revision total hip arthroplasty; THA, total hip arthroplasty; TiAlV, titanium aluminium vanadium; TiNbN, titanium niobium nitride; TJR, total joint replacement; TKA, total knee arthroplasty; TSA, total shoulder arthroplasty; UHMWPE, Ultrahigh Molecular Weight Polyethylene; UKA, unicondylar knee arthroplasty.

### Diagnostic tests

Twelve studies used patch testing to assess metal hypersensitivity [8, 9, 12, 15, 21, 22, 28, 30, 35, 36, 40, 50], while LTT was performed in nine papers [9, 18, 28, 34, 40, 45, 49, 50, 51]. Fourteen papers identified allergic patients through medical history, either alone or in combination with patch testing, LTT, or both [4, 12, 21, 22, 24, 25, 28, 35, 36, 37, 40, 43, 45, 47]. One article did not report the diagnostic test used [16].

In seven studies, patients underwent testing only postoperatively [4, 8, 9, 18, 28, 35, 40], while in ten testing was performed only preoperatively [16, 25, 34, 37, 43, 45, 47, 49, 50, 51]. In five studies the same patients were tested both preoperatively and postoperatively [12, 22, 30, 36].

One paper performed testing on one cohort of participants before surgery and on a different cohort after surgery [15].

The recorded time at which testing was performed postoperatively ranged from 6 months to 396 months, with eight studies not reporting when the postoperative testing was conducted [4, 8, 9, 18, 28, 30, 35, 40]. Two papers did not describe when hypersensitivity tests were carried out [21, 24].

Type and timing of testing are shown in Table 3.

**Table 3:** Diagnostic tools.

Author	Hypersensitivity test	Timing of testing
Bloemke et al.	Self‐reported allergy	Postoperatively (time frame NR)
Desai et al.	Patch test	Postoperatively (time frame NR)
Dietrich et al.	Patch test, LTT	Postoperatively (time frame NR)
Frigerio et al.	Self‐reported allergy, patch test	Preoperatively Postoperatively (after 1 year)
Granchi et al.	Patch test	Preoperatively (group A) Postoperatively (Group B: median follow‐up 76 months, range 12–216 months; Group C: median follow‐up 99 months, range 12–396 months)
Guenther et al.	NR	Preoperatively
Hallab et al.	LTT	Postoperatively (Group 2, time frame NR)
Kennon et al.	Self‐reported allergy, patch test	NR
Kręcisz et al.	Self‐reported allergy, patch test	Preoperatively Postoperatively (mean follow‐up 24 months ± 0.52)
Law et al.	Self‐reported allergy	NR
Lawrie et al.	Self‐reported allergy	Preoperatively
Mayer et al.	Self‐reported allergy, patch test, LTT	Postoperatively (time frame NR)
Münch et al.	Patch test	Preoperatively (253 patients) Postoperatively (74 patients, time frame NR)
Schneiderman et al.	LTT	Preoperatively
Schultzel et al.	Self‐reported allergy, patch test	Postoperatively (time frame NR)
Shanmugham et al.	Self‐reported allergy, patch test	Preoperatively Postoperatively (after 6 months)
Siljander et al.	Self‐reported allergy	Preoperatively
Summer et al.	Self‐reported allergy, patch test, LTT	Postoperatively (time frame NR)
Thienpont et al.	Self‐reported allergy	Preoperatively
Tidd et al.	Self‐reported allergy, LTT	Preoperatively
Walker et al.	Self‐reported allergy	Preoperatively
Yang et al.	LTT	Preoperatively
Zeng et al.	Patch test, LTT	Preoperatively
Zondervan et al.	LTT	Preoperatively

Abbreviations: LTT, lymphocyte transformation testing; NR, not reported.

### Outcome measures and complications

Nine studies explored postoperative outcome measures [21, 24, 37, 43, 45, 47, 49, 50, 51], though the diversity of reported metrics limits direct comparison. Four papers [21, 24, 47, 49] reported outcome measures in patients with metal allergy receiving the same implant, while in one [37] allergic patients receiving nickel‐free or standard implants were assessed.

Three studies [43, 45, 51] evaluated allergic and non‐allergic individuals undergoing procedures with nickel‐free implants and standard implants, respectively. One paper [50] included allergic and non‐allergic patients but did not record the metallic composition of the implants used.

The included studies utilised a wide range of outcome measures, making synthesis and interpretation challenging. To improve clarity, focus was placed on key functional and pain‐related metrics: ROM, Knee Society Score (KSS), KOOS, VAS for pain, and patient satisfaction. Standardising the use of these core measures in future studies would enhance comparability and clinical relevance.

Complications were reported in twelve papers [8, 9, 15, 16, 21, 24, 28, 35, 37, 40, 43, 47]. Outcome measures and complications are shown in Tables 4 and 5.

**Table 4:** Complications.

Author	Complications
Desai et al.	Pain in 6 patients Loss of function in 5 patients Patient dissatisfaction in 5 patients
Dietrich et al.	Swelling in 3 patients Pain in 3 patients Effusions in 3 patients Erythema in 2 patients Soft tissue oedema in 1 patient Early loosening in 1 patient
Granchi et al.	THA failure occured significantly earlier in patients with postivie patch testing
Guenther et al.	Allergic rections in 17 patients: malaise and nausea, itching, skin reactions and/or arthrofibrosis, early implant loosening
Kennon et al.	Self‐reported allergy group: Acromial fractures in 2 patients Glenoid loosening in 1 patient Reoperations for loose glenoid components in 2 patients Patch test group: Glenoid loosening in 1 patient
Law et al.	Residual knee stiffness in 3 patients Infection in 2 patients Instability in 2 patients Aseptic loosening in 1 patient Periprosthetic fracture in 1 patient
Mayer et al.	Swelling in 48 patients Pain in 56 patients Itching in 20 patients Burning in 12 patients Implant loosening in 19 patients Instability in 21 patients Loosening/instability combined in 29 patients Decreased range of motion in 13 patients
Schultzel et al.	Persistent oedema, erythema, postoperative joint pain in 4 patients
Siljander et al.	CoCr implant: Synovitis in 3 patients Effusion in 7 patients Nickel‐free implant: Dermatitis in 5 patients Synovitis in 21 patients Effusion in 47 patients
Summer et al.	Pain in 5 patients Reduced mobility in 3 patients Eczema in 3 patients Swelling in 2 patients Effusion in 2 patients
Thienpont et al.	Group 1 Reoperation in 3 patients: 1 for aseptic loosening, 1 for chronic pain, 1 arthrolysis Group 2 Reoperation in 4 patients: 1 for hematogenous infection, 1 for low‐grade infection, 1 for aseptic loosening, 1 complete revision
Walker et al.	Unspecific recurrent swelling in 1 patient Reoperation in 2 patients (wound dehiscence in 1 patient, suspicion of early infection in 1 patient)

Abbreiation: THA, total hip arthroplasty.

**Table 5:** Reported outcomes.

Author	Outcome measures
Kennon et al.	Self‐reported allergy group Active forward elevation: 80°–141° postoperatively Active external rotation: 24°–52° postoperatively Active internal rotation: mostly lubmosacral to mostly lumbar postoperatively Pain scores: 96% moderate‐severe to 88% non‐mild postoperatively Patch test group Active forward elevation: 84°–146° postoperatively Active external rotation: 18°–59° postoperatively Active internal rotation: mostly lumbosacral to mostly lumbar postoperatively Pain scores: 100% moderate‐severe to 92% non‐mild postoperatively
Law et al.	ROM: 109° ± 15° to 112° ± 13° postoperatively UCLA activity scale: 4.1 ± 1.5 to 5.1 ± 2 postoperatively KSP scores: 4.5 ± 8.5 to 42.9 ± 14 postoperatively KSC scores: 36 ± 145 to 89 ± 15 postoperatively KSF scores: 48 ± 145 to 73 ± 28 postoperatively
Siljander et al.	CoCr implant KOOS JR: 50.3 ± 3.0 to 72.8 ± 3.5 postoperatively PROMIS10 mental score: 52.2 ± 1.9 to 50.9 ± 5.6 PROMIS10 physical score: 42.7 ± 1.7 to 47.9 ± 4.9 postoperatively LEAS score: 8.0 ± 0.6 to 9.8 ± 0.7 postoperatively VAS score: 67.9 ± 5.3 to 24.8 ± 5.3 postoperatively VR12 mental score: 49.3 ± 2.6 to 57.4 ± 2.7 VR12 physical score: 30.3 ± 2.3 to 41.4 ± 2.4 Nickel‐free implant KOOS JR: 50.6 ± 1.1 to 74.9 ± 1.2 postoperatively PROMIS10 mental score: 50.3 ± 0.8 to 50.01 ± 1.4 postoperatively PROMIS10 physical score: 42.3 ± 0.7 to 47.1 ± 1.2 postoperatively LEAS score: 8.9 ± 0.2 to 10.4 ± 0.3 postoperatively VAS score: 64.6 ± 1.9 to 17.4 ± 2.1 postoperatively VR12 mental score: 54.3 ± 0.9 to 55.4 ± 0.9 postoperatively VR12 physical score: 29.1 ± 0.8 to 41.7 ± 0.8 postoperatively
Thienpont et al.	Group 1 Postoperative KSS: 75 ± 20 Postoperative KOOS: 80 ± 22 Postoperative FJS‐12: 68 ± 32 Flexion: 119° ± 13° to 125° ± 12° postoperatively Extension: ‐3° ± 5° to 0° ± 2° postoperatively Group 2 Postoperative KSS: 70 ± 20 Postoperative KOOS: 79 ± 19 Postoperative FJS‐12: 58 ± 34 Flexion: 117° ± 16° to 128° ± 10° Extension: −5° ± 6° to 0° ± 3°
Tidd et al.	Hypoallergenic group: LOS: 1.6 ± 1.03 days KOOS pain: 39.3 ± 15.9 to 81.9 ± 16.9 postoperatively KOOS PS: 47.6 ± 15.8 to 74.8 ± 13.5 postoperatively VR‐12 MCS: 50.5 ± 12.0 to 52.8 ± 9.8 postoperatively Standard group: LOS: 1.6 ± 0.93 days KOOS pain: 39.7 ± 16.7 to 82.1 ± 18.8 postoperatively KOOS PS: 47.9 ± 16.5 to 75.0 ± 15.7 postoperatively VR‐12 MCS: 50.4 ± 12.4 to 52.7 ± 10.2 postoperatively
Walker et al.	Postoperative OKS: 42.5 ± 2.5 Satisfaction with the prosthesis: 0.61 ± 0.71
Yang et al.	KSS clinical score: 49.8 ± 16.7 to 75.6 ± 14.6 postoperatively KSS functional score: 44.8 ± 16.7 to 57.4 ± 15.5 postoperatively ROM: 93.2° ± 25.2° to 116.6° ± 19.9° postoperatively
Zeng et al.	THA group VAS score in metal allergy group: 4.14 ± 0.99 to 0.39 ± 0.80 postoperatively VAS score in non‐metal allergy group: 4.27 ± 1.54 to 0.38 ± 0.57 postoperatively TKA group VAS score in metal allergy group: 5.00 ± 0.45 to 0.90 ± 0.54 postoperatively VAS score in non‐metal allergy group: 4.71 ± 2.02 to 0.86 ± 0.66 postoperatively
Zondervan et al.	Reactive group PI‐NRS: 7.15 ± 2.06 to 3.91 ± 2.84 postoperatively ROM: 94.74° ± 27.98° to 112.19° ± 16.01° postoperatively Non‐reactive group: PI‐NRS: 5.71 ± 2.63 to 1.25 ± 1.77 postoperatively ROM: 90° ± 17.83° to 111° ± 15.56°

Abbreviations: FJS‐12, forgotten joint score; KOOS, knee osteoarthritis outcome score; KSC, Knee Society Clinical; KSF, Knee Society Functional; KSP, Knee Society Pain; LEAS, Lower Extremity Activity Scale; LOS, length of stay; OKS, Oxford Knee Score; PI‐NRS, Pain Intensity Numerical Rating Scale; PROMIS10, Patient‐Reported Outcomes Measurement Information System; ROM, range of motion; THA, total hip arthroplasty; UCLA, University of California; VAS, Visual Analog Scale; VR12, Veterans RAND 12‐item Component Survey.

## DISCUSSION

The main findings of this review highlight ongoing disagreements regarding the most effective diagnostic methods for assessing metal hypersensitivity in patients undergoing joint arthroplasty [46]. The lack of consistency in protocols‐ where some practitioners rely on patch testing, others on LTT, and still others on patient history‐ can significantly influence clinical decision‐making, particularly when choosing between a nickel‐containing prosthesis and a nickel‐free alternative. This lack of standardised protocols introduces uncertainty, potentially impacting patient outcomes and care [12, 19]. None of those tests are reliable, sensitive and specific enough to base treatment decisions on them [13, 32]. A metal hypersensitivity should only be diagnosed after exclusion of all other reasons of failure [1]. Given the absence of a standardised protocol, clinicians should adopt a multifaceted approach that considers patient history, clinical symptoms, and available diagnostic tools [33]. While no definitive test exists, combining patch testing with lymphocyte transformation tests may provide greater diagnostic accuracy [32, 49]. Clinicians should also engage in shared decision‐making with patients regarding implant selection [33].

Of the 24 studies reviewed, twelve reported complications such as joint pain and swelling, reduced range of motion, eczematous reactions, and even implant failure [3, 42]. However, it remains unclear whether all these complications are directly related to metal hypersensitivity [20].

The prevalence of metal hypersensitivity, particularly to nickel, followed by chromium and cobalt, is notably higher in patients with orthopedic implants compared to the general population [23]. This increased prevalence is especially pronounced in patients with poorly functioning or failed implants, aligning with previous studies that suggest a potential link between metal hypersensitivity and implant failure [17, 48]. Despite the frequent use of skin patch testing as a diagnostic tool, the review emphasises the ongoing debate over its reliability, particularly in predicting postoperative complications [32]. Given the inconsistent findings across studies, the hypothesis that skin patch testing is a reliable predictor of postoperative complications is rejected. The lack of standardisation and variability in test results further undermine its clinical utility [32, 33]. Several studies focused on the limitations of patch testing in the orthopedic setting [29, 41]. Similarly, while LTT has shown promise [32], its widespread adoption is impeded by issues such as lack of standardisation and variability between laboratories [15, 17]. Fourteen studies identified allergic patients based on medical history, either alone or in conjunction with patch testing, LTT, or both [4, 12, 21, 22, 24, 25, 28, 35, 36, 37, 40, 43, 45, 47].

A significant issue identified in this review is the variability in the timing of hypersensitivity testing. Some studies conducted testing preoperatively, others postoperatively, and a few performed both, leading to inconsistent reports on the prevalence of metal sensitivity and its clinical impact. The timing of testing is crucial, as hypersensitivity may develop or become more pronounced after implantation due to ongoing exposure to metal ions [14]. This variation may contribute to the unreliable findings regarding the impact of metal hypersensitivity on clinical outcomes [22]. The inconsistency suggests that a standardised approach to testing timing could provide more definitive insights into the role of metal hypersensitivity in joint arthroplasty success [14].

Clinical outcomes in patients with metal hypersensitivity varied widely among the studies reviewed. While some research reported no significant differences between hypersensitive and non‐hypersensitive patients [43], suggesting that hypersensitivity may not always lead to clinical symptoms or implant failure [6], others indicated that hypersensitivity might be associated with increased pain, reduced joint function, and lower overall patient satisfaction [3, 42, 49, 51]. Particularly, one article [22] reported that three patients developed a positive reaction to nickel post‐operatively and experienced periodical skin lesions, pain, swelling and erythema. Another study documented persistent oedema, erythema, and postoperative joint pain in four patients [35].

Although less common, systemic reactions such as dermatitis or systemic allergic reactions have been pointed out. These reactions may involve symptoms like itching, rashes, or more severe allergic responses throughout the body [16, 28]. Overall, reoperation rates in patients with metal hypersensitivity may be influenced by a combination of factors including the type of implant, the extent of hypersensitivity reactions, and the effectiveness of initial surgical and diagnostic interventions [14, 48].

These mixed findings reflect the complexity of the issue and suggest that metal hypersensitivity may contribute to implant failure in some cases but not others, potentially due to variations in individual immune response, surgical techniques or implant materials. Moreover, patient‐specific factors like comorbidities and genetic predispositions, also play critical roles [13, 44].

Nickel‐free prostheses are particularly crucial for individuals with a diagnosed nickel allergy or hypersensitivity, which can cause allergic reactions ranging from mild dermatitis to severe systemic responses upon contact with nickel‐containing materials [10]. Nickel‐free alternatives are designed to address this problem by using materials that do not contain nickel, thus reducing the risk of adverse reactions and associated complications compared to traditional nickel‐containing prosthetics [10]. Materials such as titanium, which is biocompatible and free from nickel, are often employed in these prostheses. Additionally, cobalt‐chromium alloys and high‐grade polymers have been developed to provide durable and allergy‐free options [13]. Overall, the adoption of nickel‐free prostheses represents a significant advancement in prosthetic technology, offering enhanced safety, longevity, comfort, and performance for patients with nickel allergies and sensitivities [7, 10, 13, 26].

The present paper systematically reviews a broad range of studies on metal hypersensitivity in joint arthroplasty, providing an important overview of the current state of knowledge on this topic.

The inclusion of multiple databases and the examination of various study designs (prospective, retrospective, case–control and case series) enhances the strength and reliability of the findings. Case control studies were judged as “low risk of bias” or “moderate risk of bias”. The mean quality of case‐series studies was overall good.

However, this systematic review has several limitations. The included studies are retrospective case series and retrospective case‐control studies with low level of evidence. These study designs limit the strength of the conclusions drawn from the review. The review also lacks a substantial number of prospective, randomised controlled trials, which are the gold standard for establishing causality, reducing the overall level of evidence and limits the ability to make definitive recommendations. Future research should focus on defining patient risk profiles, refining diagnostic tools, and evaluating long‐term outcomes to guide standardised management strategies. Moreover, the variability in the timing of hypersensitivity testing across studies represents another limitation, as it complicates the ability to compare results and establish consistent patterns or recommendations. Furthermore, this review does not extensively explore other patient‐specific factors (e.g., genetic predispositions and comorbidities) that could influence the relationship between hypersensitivity and implant outcomes. Finally, the studies included in the review report a wide variety of clinical outcomes, such as pain scores, ROM, and patient satisfaction. However, not all studies use the same measures or report them consistently, making it challenging to compare results across studies.

## CONCLUSION

There is a notable absence of a standardised protocol for diagnosing metal hypersensitivity, both in terms of which tests to perform and the timing of these tests. Additionally, there is significant variability in the reported outcomes, with few studies focusing on patients with hypersensitivity both before and after surgery. This lack of standardisation and comprehensive pre‐operative and post‐ operative analyses limits the understanding of complications related to hypersensitivity, thereby affecting clinical decision‐making regarding the choice of prosthesis. Clearly, a metal hypersensitivity should only be diagnosed after exclusion of all other reasons of failure. However, clinicians can adopt interim strategies based on current evidence. There is a clear need for continued research and collaboration in refining diagnostic and treatment strategies for metal hypersensitivity in joint arthroplasty.

## AUTHOR CONTRIBUTIONS

*Conceptualisation*: Umile Giuseppe Longo and Michael T. Hirschmann. *Methodology*: Giovanni Intermesoli and Alberto Lalli. *Software*: Bruno Violante. *Validation*: Umile Giuseppe Longo and Michael T. Hirschmann. *Formal analysis*: Raffaele Di Tommaso and Alberto Lalli. *Investigation*: Giovanni Intermesoli and Alberto Lalli. *Data curation*: Bruno Violante. *Writing—original draft preparation*: Raffaele Di Tommaso, Giovanni Intermesoli, and Alberto Lalli. *Writing—review and editing*: Umile Giuseppe Longo. *Visualisation*: Bruno Violante. *Supervision*: Umile Giuseppe Longo. *Project administration*: Michael T. Hirschmann and Umile Giuseppe Longo. All authors have read and agreed to the published version of the manuscript.

## CONFLICT OF INTEREST STATEMENT

The authors declare no conflicts of interest.

## ETHICS STATEMENT

The Institutional Review Board of Campus Bio‐Medico University of Rome ruled that no formal ethics approval was required in this particular case. The access to the database is on request. The author(s) read and approved the final article.

## Data Availability

All data are available upon request to the corresponding author.
